# Effective Viscoplastic-Softening Model Suitable for Brain Impact Modelling

**DOI:** 10.3390/ma15062270

**Published:** 2022-03-18

**Authors:** Bartłomiej Dyniewicz, Jacek M. Bajkowski, Czesław I. Bajer

**Affiliations:** 1 Institute of Fundamental Technological Research, Polish Academy of Sciences, Pawińskiego 5b, 02-106 Warszawa, Poland; 2 Faculty of Mechanical and Industrial Engineering, Warsaw University of Technology, Narbutta 85, 02-524 Warszawa, Poland

**Keywords:** brain biomechanics, brain injury, mechanical properties of brain tissue, viscoplastic materials, numerical modelling, finite element method (FEM), dynamic response, acceleration, space–time FEM

## Abstract

In this paper, we address the numerical aspects and implementation of a nonlinear viscoplastic model of the mechanical behaviour of brain tissue to simulate the dynamic responses related to impact loads which may cause traumatic injury. Among the various viscoelastic models available, we deliberately considered modifying the Norton–Hoff model in order to introduce non-typical viscoplastic softening behaviour that imitates a brain’s response just several milliseconds after a rapid impact. We describe the discretisation and three dimensional implementation of the model, with the aim of obtaining accurate numerical results in a reasonable computational time. Due to the large scale and complexity of the problem, a parallel computation technique, using a space–time finite element method, was used to facilitate the computation boost. It is proven that, after calibrating, the introduced viscoplastic-softening model is better suited for modelling brain tissue behaviour for the specific case of rapid impact loading rather than the commonly used viscoelastic models.

## 1. Introduction

Accurate modelling of the mechanical properties of brain tissue becomes of high importance when designing head protection systems to mitigate the effects of rapid excitation by reducing the amplitude of acceleration and shortening the duration of the harmful impact. Moreover, specific models may be used as predictive tools in forensic medicine, as well as in helping to plan ahead after trauma treatment or surgical operations [1,2,3].

In recent decades, scholars have proposed multiple brain models to account for particular features of brain tissue response under specific loading conditions and testing regimes [4]. Various constitutive material models suited for a particular type of brain tissue analysis are often referred to in the literature and are still developed [5,6,7,8,9,10,11]. Many studies have shown that soft tissues exhibit properties that are difficult to describe with rheological relations, assuming linear elasticity or viscoelasticity, because their characteristics may be highly nonlinear [12,13,14]. This discrepancy is especially important for studies concerning the modelling of a protected or unprotected head impacting a hard obstacle, and was the core motivation for introducing a new, more reliable model, as discussed in this paper.

The main aim of our study was to develop a numerical model that accurately mimics the actual mechanical behaviour of the brain’s soft tissue during the first few milliseconds after a human head hits against a hard, flat surface. The accurate modelling of this particular stage of the response is crucial to predicting dangerous acceleration peaks and identifying regions that are most vulnerable to injury. Among the various viscoelastic models available, we have used the viscoplastic Norton–Hoff model and introduced a non-typical strain-rate softening behaviour with negative rate sensitivity *m* and coefficient *s*, responsible for adjusting the hyperbolic shape of dependency. The parameters of the model were calibrated based on the results from reference literature [15].

Because accurate finite element simulations of the human brain require high computational power, we used a simplified head model for initial optimisation, and then a higher resolution model to obtain acceleration maps for the first few milliseconds after the impact. Due to the large scale of the problem, we implemented a custom space–time finite element method (FEM) adapted for parallel code execution, which allowed us to obtain results almost 25 times faster than using standard finite elements and serial code execution.

The outline of the paper is as follows. In Section 2.1, a brief overview of the issues related to brain modelling during rapid head impacts are discussed; the reasoning for choosing a viscoplastic model is justified. Section 2.2 addresses the mathematical modelling effort, as well as the numerical implementation of the viscoplastic model, and recalls the basics of space–time FEM modelling. In Section 3.1, parameter identification is discussed along with limitations of the selected approach. The numerically obtained acceleration maps for a full scale brain model, computed using parallel code processing, are presented in Section 4. Finally, Section 5 presents the conclusions and points out the main benefits guiding the effort for future model refinements.

## 2. Materials and Methods

### 2.1. Beyond the Viscoelastic Model

First of all, we need to consider the modelling problem of a brain hitting a rigid obstacle during an impact or a fall. A vast group of studies use basic models that only consider the linear elastic modulus, assuming that the mechanical behaviour of the soft tissue is independent of the loading rate. While easier to compute, the common viscoelastic models do not accurately replicate mechanical behaviour, because they are characterised by an oscillating response to disturbance during the first phase of motion after impact while the sustained oscillating motion is gradually dissipated [16,17].

**Figure 1:**
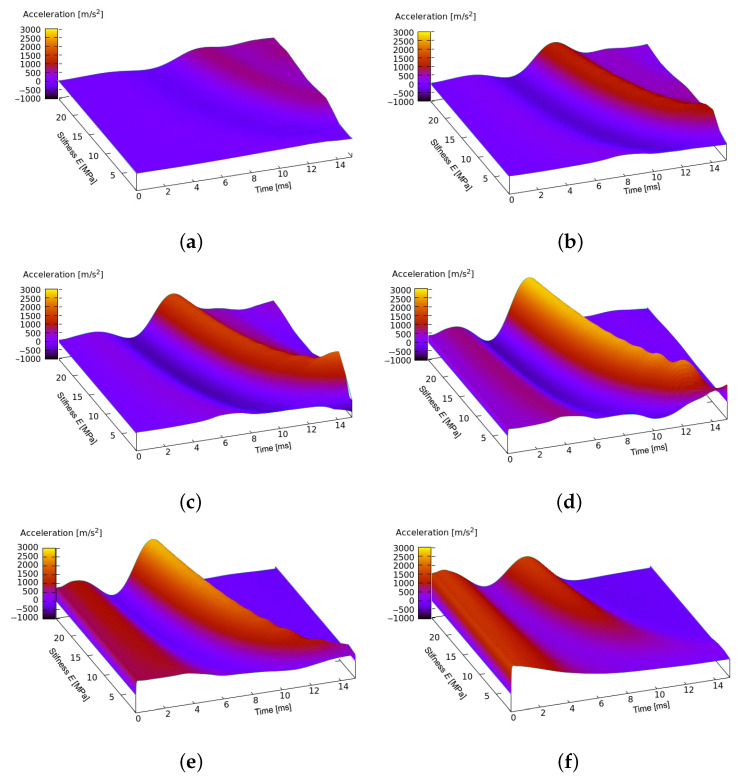
Simulation results for linear viscoelastic material with constant damping of (**a**) 1 Ns/m, (**b**) 5 Ns/m, (**c**) 10 Ns/m, (**d**) 50 Ns/m, (**e**) 100 Ns/m, and (**f**) 200 Ns/m.

Figure 1 presents exemplary numerical results for linear viscoelastic material, described with viscous damping *c* up to 200 Ns/m and stiffness *E* up to 25 MPa, hitting a rigid obstacle. Each graph shows this relation as a 3D surface for different damping values, i.e., 1, 5, 10, 50, 100, 200 Ns/m. For low damping values up to 50 Ns/m, the acceleration reaches up to 3000 m/s${}^{2}$. One can see auxiliary hardening behaviour for damping above 50 Ns/m, as presented in Figure 1d–f. This immediately demonstrates that after the impact there is increased acceleration, which then gradually diminishes.

**Figure 2:**
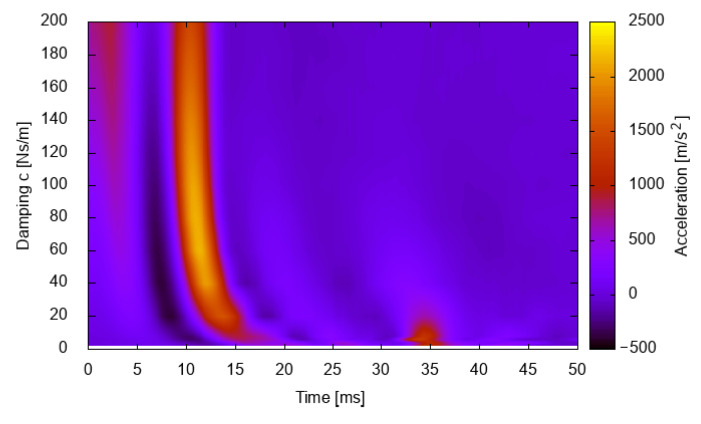
Simulation results for linear viscoelastic material with constant stiffness of *E* = 25 MPa.

In all cases, rising stiffness increases the wave speed and, thus, the acceleration starts to build up with a 9 ms delay for high stiffness. This delay results from the time required for the wave to travel from the point of contact with the obstacle to the measuring point, and is an obvious drawback for a viscoelastic material, which is not observable in real-life experiments on brain impacts. Moreover, the time delay is also influenced by damping. The data concerning the first 50 ms after the impact, presented in Figure 2, are consistent with Figure 1. However, a different presentation method is used. A constant stiffness of 25 MPa is assumed. The damping values are now presented on the vertical axis, so the resolution is much greater than the gradual increase used beforehand. The response delay is clearly noticeable. The acceleration wave response reaches a maximum after 9 ms for high damping values. However, the maximum is reached after 11–13 ms for lower damping values. This lag is immanent for the viscoelastic model but does not correspond to the experimental results [15,18] that report an immediate acceleration increase after hitting the obstacle, without any significant delay.

The initially strong hardening related to high damping (a significant delay in the acceleration wave), as well as a too rapid decrease of acceleration (when returning back to equilibrium), are not observable in real-life experiments concerning the brain-tissue response, and are the main downsides of such models. On the basis of these observations, the authors of this study argue that more accurate modelling of the response of brain tissue in the phase just after the impact can be achieved using material similar to a dense liquid or soft plastic matter with a high viscosity coefficient, rather than a viscoelastic one. While “in vitro” studies suggest the elasticity of brain tissue, these properties are typically exhibited in the quasi-static range. Thus, a nonlinear viscoplastic model seems to be better suited to the numerical modelling of brain tissue subjected to short-term acceleration or deceleration.

In [14], the authors showed that standard linear elastic models do not accurately reflect the viscoelastic properties of porcine, soft brain tissue, since the stiffness depends on the loading rate. In [19], the authors emphasised problems with the assumptions of linear elasticity of soft brain tissue when large strains and discontinuities are present. To model the force of the interaction between the biopsy needle and the brain tissue, the authors in [20] used viscoelastic plastic properties and considered the relaxation behaviour of the material. In [21], the authors presented a model for brain-like tissue mechanics, showing that rat livers exhibit shear strain softening and compression stiffening. The nonlinear viscoelastic behaviour of the cerebral structure was established in [22] and used in many studies considering large deformations and, thus, large strains on the brain structure [12,23,24]. In [25], the study on wet and dry porcine brains undergoing quasi-static and high strain rate mechanical deformations revealed that the hydration level plays a significant role in tissue micromechanics. The stress levels for the dry brain were more significant than the wet brain, while the dry brain stress–strain behaviour was similar to ductile materials with a yield point and work hardening. However, the wet specimen revealed a concave inflexion typical for polymers. Some of the extended models introduce hyperelastic and hyper-viscoelastic behaviour, or the combined features of basic models [8,17,26,27,28,29]. In [30], the authors modelled a brain’s material properties using both hyperelastic and viscoelastic constitutive laws. Some more advanced models for soft tissues include plasticity, hysteresis, and biphasic response [31,32,33,34].

The modelling of rapid head impacts requires accounting for large strains and time and rate effects. Thus, standard brain models accounting for small perturbations fail to deliver valuable results [35,36]. In [37], four visco-hyperelastic models were employed for the simulation of traumatic brain injuries and the experimental results were compared. Some models gave inaccurate or even erroneous results, with a polynomial model being the most accurate. To simulate damage to the soft tissues through inelastic deformations due to frontal and oblique head impacts with external objects, the authors in [38] included rate effects, shear, porous plasticity, and finite viscoelasticity. The selection of the constitutive model for a given application depends on the strain rate of the process and, to a certain extent, on the required computational efficiency. In [39], the authors recommended avoiding linear models and using nonlinear models instead. In [40] the Ogden hyperviscoelastic, Mooney–Rivlin hyperviscoelastic, neo–Hookean hyperviscoelastic, and linear viscoelastic constitutive models were used to compare their applicability to model the brain tissue under the violent impact. The study showed that applying the linear viscoelastic model may lead to overestimating deformations within the brain. For more nuances, we refer the reader to [41], which covers the 184 latest publications on the mechanical testing and modelling of brain tissue.

Each of the modelling approaches aims to account for essential features of the tissue response under selected test conditions, but integrating all the characteristic features of each model into one single constitutive framework seems impractical. The determination of a reliable model incorporating plasticity remains an open issue for experimenters and numerical researchers. Therefore, the main motivation of the present research is to verify the viability of the modified Norton–Hoff viscoplastic modelling procedure, rather than the clinical verification of biomechanical parameter results.

### 2.2. Space–Time Viscoplastic Model

When assembling a material model to be used for dynamic simulation, one needs to consider the complexity of multi-parameter identification and the extensive computation power required to run simulations. In this paper, a uniform, isotropic and noncompressive material, described by a relatively simple Norton–Hoff formula for a viscoplastic solid [42], was chosen for investigation. The model itself is straightforward and convenient to use, as it only requires a limited number of parameters to be identified experimentally. The nonlinear model was used and later modified by introducing rate-dependent softening and an additional modifier, to achieve better compatibility with the reference results.

The space–time description of the problem was used, since the evolution of the geometry during plastic flow is more convenient with simultaneous interpolation continuing in space and time. Moreover, the space–time FEM facilitates parallel computing and, when properly adapted, allows the results to be obtained much quicker than in classical FEM. Since the full formulation of the space–time approach can be found in [43], only the outline is recalled below.

The problem is considered in space x and time *t*. The space–time interpolation of unknowns ${\tilde{q}}_{e}$ is continued with the use of shape functions $\tilde{N}\left(x,t\right)$ based on node parameters in the space–time finite element

(1) $$u\left(x,t\right)=\tilde{N}\left(x,t\right)\;{\tilde{q}}_{e}\;,$$

instead of a classical finite element interpolation

(2) $$u\left(x,t\right)=N\left(x\right)\;q_{e}\times T\left(t\right)\;.$$

**Figure 3:**
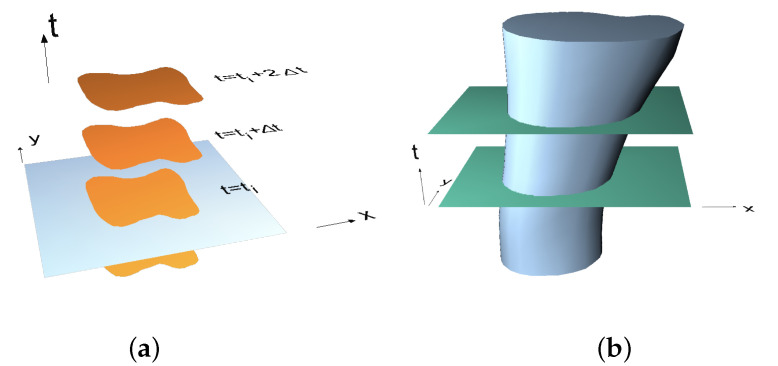
Exemplary 2D object in a classical approach (**a**) and evolving in space–time (**b**).

In the classical approach (2), the interpolation is carried on in a chosen discrete time $t_{i}$, $t_{i+1}$ etc., and is extended over time, for example, with the Runge-Kutta or Newmark method, resulting in a discontinuous representation of both unknowns and geometry (Figure 3a). Instead, Equation (1) allows the continuous approximation in time, and thus continuous space–time modelling (Figure 3b).

If we define ε=Du, where D is the differential operator, u is a displacement vector, and stress σ=Eε, where E is the elasticity matrix, the equation of virtual work will have the form

(3) $$\int_{\Omega }{\left(v^{*}\right)}^{T}\rho \;\frac{\partial v}{\partial t}d\Omega +\int_{\Omega }{\left({\dot{\varepsilon }}^{*}\right)}^{T}\sigma d\Omega +\int_{\Omega }{\left(v^{*}\right)}^{T}c\;v\;d\Omega =\int_{\Omega }{\left(v^{*}\right)}^{T}fd\Omega \;,$$

where v and $v^{*}$ are real and virtual velocity, respectively. Ω is the space–time volume and can be written as Ω=V(t)×[0;t], where *V* is the spatial volume varying in time interval [0;t]. ρ is the mass density, *c* is the damping coefficient and f expresses external forces.

Displacement u is written as the integral $u\left(t\right)=u_{0}+\int_{0}^{t}\;v\;dt,$ where $u_{0}$ is the displacement vector at the beginning of the time step. It is assumed that the displacement at the end of the proceeding time interval is computed as the integral of the velocity over the time step. Taking the above relationships into account, the equation of virtual work (3) has the form

(4) $$\begin{array}{c} \int_{\Omega }{\left(v^{*}\right)}^{T}\rho \;\frac{\partial v}{\partial t}d\Omega +\int_{\Omega }{\left(Dv^{*}\right)}^{T}EDu_{0}d\Omega +\int_{\Omega }\left[{\left(Dv^{*}\right)}^{T}\;E\;D\;\;\int_{0}^{t}\;v\;dt\right]d\Omega +\\ \int_{\Omega }{\left(v^{*}\right)}^{T}c\;v\;d\Omega =\int_{\Omega }{\left(v^{*}\right)}^{T}fd\Omega \;. \end{array}$$

N and $N^{*}$ are shape functions for the interpolation of the real and virtual velocities, respectively. Because functions are integrated over the space–time subdomain, the interpolation must be carried out from nodal parameters in time $t_{i}$ and $t_{i+1}$. Therefore, interpolation matrices have twice as many columns as classical spatial interpolations. Interpolations of real and virtual velocities can be written as

(5) $$v=N\dot{q}\;\;\;\mathrm{and}\;\;v^{*}=N^{*}\dot{q}\;.$$

Finally, the discrete equilibrium equation has the following form:

(6) $$\begin{array}{c} \left\{\int_{\Omega }\;\left[{\left({DN}^{*}\right)}^{T}\;E\;D\;\;\int_{0}^{t}N\;dt\right]d\Omega +\int_{\Omega }{\left(N^{*}\right)}^{T}\rho \;\frac{\partial N}{\partial t}\;d\Omega +\int_{\Omega }{\left(N^{*}\right)}^{T}c\;N\;d\Omega \right\}\dot{q}+\\ \int_{\Omega }{\left({DN}^{*}\right)}^{T}\;E\;DNq_{0}\;d\Omega =\int_{\Omega }{\left(N^{*}\right)}^{T}fd\Omega \;. \end{array}$$

In the standard Norton–Hoff model, the deviatoric part of the Cauchy stress tensor $\tau^{\prime }$ is described as follows:

(7) $$\tau^{\prime }=\frac{K\dot{\varepsilon }}{{\left(\sqrt{3}D\right)}^{1-m}},$$

where

(8) $$\dot{\varepsilon }=\frac{1}{2}\left(\mathrm{grad}\;v+{\mathrm{grad}}^{T}v\right)=Dv,$$

(9) $$D=\sqrt{\frac{2}{3}{\dot{\varepsilon }}^{T}\;\dot{\varepsilon }}.$$

**Figure 4:**
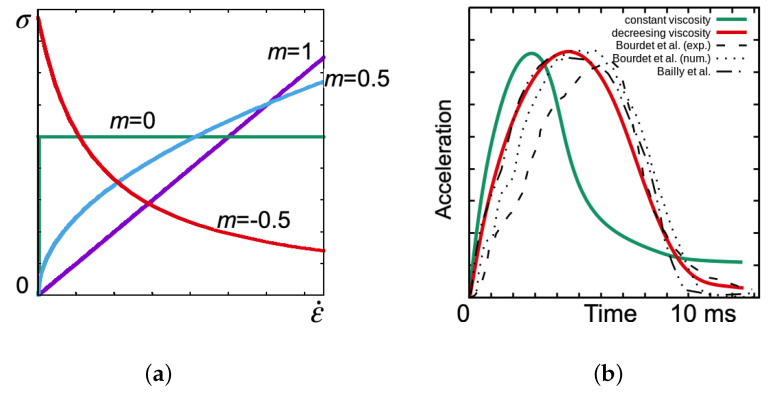
Norton–Hoff material response for different rate sensitivity *m* (**a**) and the resulting acceleration over time, compared with reference literature results [15,18] (**b**).

*K* is the material viscosity and 0≤m≤1 is the sensitivity of the deformation rate, while *m* = 0 characterises a perfectly plastic material and *m* = 1 corresponds to a Newtonian fluid, as presented in Figure 4a.

The limitation of positive values of *m* does not allow fair agreement between the numerical results and those in the literature [15]. Therefore, the model was modified by allowing negative values of −1≤m≤0, resulting in the weakening shown in Figure 4a. The viscosity decreased under the influence of increasing deformation rates and is a feature usually attributed to some biological fluids, jelly and gel-like materials [44]. The generalised material law (7)–(9) is finally proposed in a modified form:

(10) $$\tau^{\prime }=s\;\frac{K\dot{\varepsilon }}{{\left(\sqrt{3}D\right)}^{1-m}},$$

with permissible

(11) $$m\in \langle -1,1\rangle .$$

Viscosity *K*, damping *c*, and *m* can be adjusted, along with the coefficient *s*, introduced to allow different hyperbolic shapes of the dependency. Through this expansion, specific viscoplastic softening characteristics can be obtained.

When comparing the material response for different values of *m* (Figure 4b), it can be seen that assuming m<0 (softening, decreasing viscosity) gives a quick initial peak of acceleration and allows for a smooth decrease, which closely resembles the reference results.

The virtual work in a space–time layer is expressed by the integral

(12) $$\int_{0}^{h}\int_{V}{\left(v^{*}\right)}^{T}\rho \;\frac{\partial v}{\partial t}d\Omega +\int_{0}^{h}\int_{V}{\left({\dot{\varepsilon }}^{*}\right)}^{T}\tau^{\prime }d\Omega =\int_{0}^{h}\int_{\partial V}{\left(v^{*}\right)}^{T}\;f\;d\left(\partial V\right)\;dt,$$

where *h* is the considered time step. Considering the second term in Equation (12), interpolation of the virtual velocities used in Equation (8) and the real velocities in Equation (10) allow the writing of this term in the form:

(13) $$s\int_{\Omega }{\dot{q}}^{T}{\left(DN^{*}\right)}^{T}\frac{K}{{\left(\sqrt{3}D\right)}^{1-m}}DN\;d\Omega \;\cdot \dot{q}.$$

After some transformations, the matrix corresponding to this term and related to the potential energy will have the form:

(14) $$K=\int_{0}^{h}\int_{V(t)}\;{\left(DN^{*}\left(x,t\right)\right)}^{T}E\;DN\left(x,t\right)dV\;dt.$$

The analogy with the elastic case should be noted. The matrix E in the viscoplastic elements now depends on the strain rate. The remaining matrices can be derived in the same way and the final equation gains the following matrix form:

(15) $$\left(K+M+Z\right)\dot{q}=F.$$

The matrix K corresponds to the stiffness, M describes the contribution of inertia, Z contains the effect of external damping, and F is the external load vector. The incompressibility condition

(16) divv=0

which is a part of the penalty function, can be easily developed into an appropriate formula. The penalty function term charging the functional has the following form:

(17) $$\frac{1}{2}\lambda \;\mathrm{div}\;v^{*}\;\mathrm{div}\;v=\frac{1}{2}{\dot{q}}^{T}\int_{\Omega }{\left(DN^{*}\right)}^{T}\Lambda DNd\Omega \cdot \dot{q},$$

where λ is the coefficient of the penalty function and the matrix Λ, in three-dimensional problems, has the form:

(18) $$\Lambda =\lambda \left[\begin{array}{cccccc} 1 & 1 & 1 & 0 & 0 & 0\\ 1 & 1 & 1 & 0 & 0 & 0\\ 1 & 1 & 1 & 0 & 0 & 0\\ 0 & 0 & 0 & 0 & 0 & 0\\ 0 & 0 & 0 & 0 & 0 & 0\\ 0 & 0 & 0 & 0 & 0 & 0 \end{array}\right].$$

## 3. Results

### 3.1. Parameter Identification

During identification, the viscosity and damping coefficients were chosen so that the response of the modelled system corresponds well with the reference curves [15], obtained at different velocities of the head impacting the ground (of different stiffnesses).

The identification problem can be described as the search for the parameters *c* and *K* at assumed *m* that minimise the objective functional defined in point $x_{m}$ which, in our case, is the occipital part of the head:

(19) $$J=\int_{0}^{T}{\left[a\left(x_{m},t\right)-a_{d}\left(x_{m},t\right)\right]}^{2}\;dt\;.$$

*a* is the acceleration in time at a selected point, and $a_{d}$ is a function of acceleration in time at a selected point in the brain where the reference characteristic is known.

The solution of the minimisation can be written as follows:

(20) $$\mathrm{Find}\;\;\;\;\left(K,c\right)={\mathrm{argmin}}_{\begin{array}{c} K\in [K_{\min},K_{\max}]\\ c\in [c_{\min},c_{\max}] \end{array}}\;\;J\;.$$

The given task is non-linear and requires the construction of characteristic matrices of the model at each calculation step. In a dynamic process carried out with a very small time step, the computational cost of a single case increases. The minimisation of each separate set of parameters *K* and *c* and the large number of tasks comprising the graphic map increase the computational cost even more.

**Figure 5:**
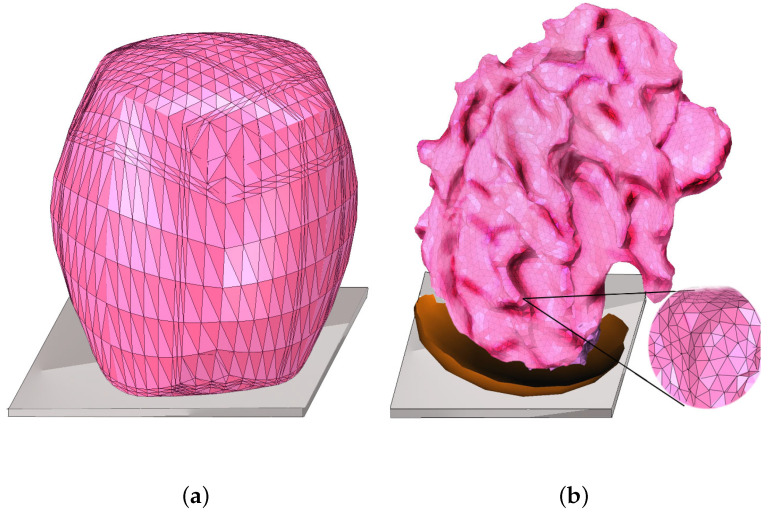
Simplified model of a head used for minimisation (**a**) and a full-scale, meshed model used for accurate acceleration mapping (**b**).

Initial calculations were made for a head model with the elastic frontal layer of the skull geometrically simplified to an ellipsoid-like shape (Figure 5a), with 14,600 d.o.f. and 21,384 elements. After obtaining optimised parameters, the ellipsoid was later replaced with a more complicated model (Figure 5b) to obtain accurate acceleration mappings.

During the impact, the front surface of the brain tissue comes into contact with the surface of the skull. In addition, the work is concerned with the response to a direct, rather than an oblique, blunt impact with a flat obstacle, and rotational effects are assumed to be negligible. Initially, the interest is focused on the global impact response characteristics, particularly the acceleration, impact severity, and duration of impact.

Initially, rate sensitivity *m* was roughly set to 0.3, 0.5, 0.7, and 0.9 in successive minimisation attempts. The experimental results of the brain impacting a solid surface with the velocity of 7.5 m/s and hitting a solid surface (an equivalent of a free fall from 2.87 m) were used as the reference curve. However, the coincidence with reference literature data was relatively poor.

**Figure 6:**
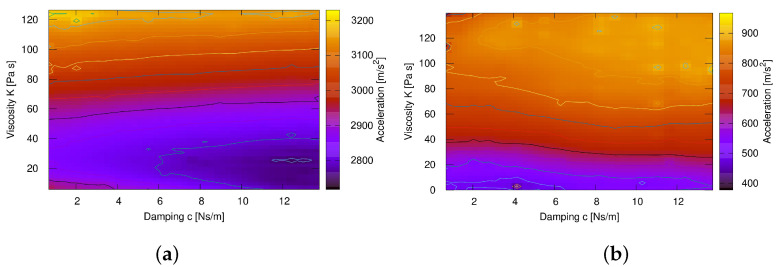
Optimum stiffness *K* and damping *c* for viscoplastic material with *m* = 0.5 (**a**) and softening with *m* = −0.5 (**b**).

Figure 6a shows the solution of optimisation for m=0.5, which gave the best results. It can be seen that the damping *c* has less influence on the acceleration than the viscosity *K*. The best parameters for viscoplastic material with *m* = 0.5 are *c* = 12 Ns/m and *K* = 30 Pa s. Acceleration for such a case reaches 2750 m/s${}^{2}$, which strongly exceeds the maximum peak of 800–1400 m/s${}^{2}$ from the results in the literature, for different cases considered [15].

The optimisation results obtained for softening characteristics were obtained for softening sensitivity *m* set to −0.3, −0.5, −0.7, and −0.9. The best agreement with the reference results was obtained at m=−0.5, with *c* = 12 Ns/m and *K* = 20 Pa s (Figure 6b). At this stage, coefficient *s* was assumed to be 1. The optimised parameters allowed to achieve an acceleration of 800–1400 m/s${}^{2}$, which fits the reference results.

## 4. Simulation and Acceleration Mapping

**Figure 7:**
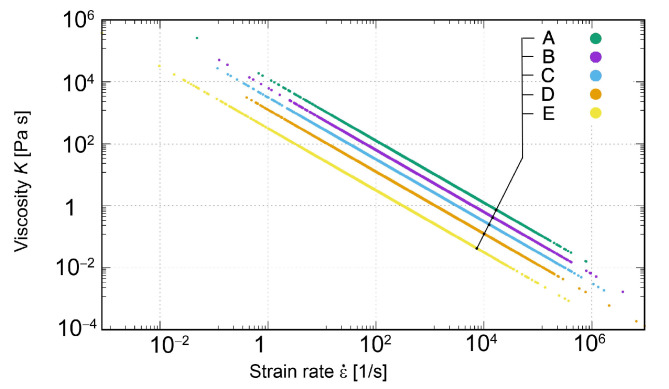
Relation between viscosity *K* and the strain rate $\dot{\varepsilon }$ for softening matter.

The assumed variants of material parameters for softening characteristics at m=−0.5 and values of coefficient *s* are presented in Table 1. The relation between viscosity *K* and the strain rate $\dot{\varepsilon }$, for cases of softening matter A–E, is depicted in Figure 7.

**Figure 8:**
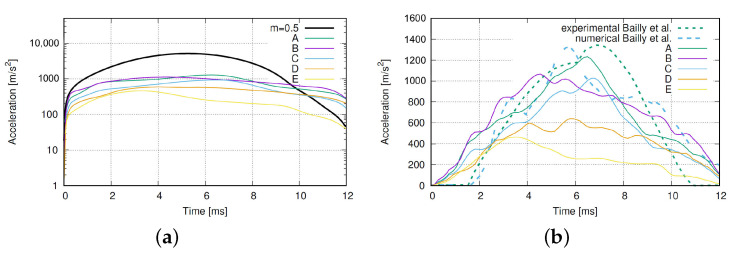
Acceleration in time for: (**a**) Constant parameters at m=0.5 and softening at m=−0.5 and (**b**) the expanded comparison of simulation and with literature results [15].

The acceleration over time, for m=−0.5, is depicted by the thick line in Figure 8, while other lines represent the results for weakening cases A–E. The view is expanded for better clarity in Figure 8b. It compares reference results [15] with simulations of the viscoplastic softening model. The proposed model achieves fair agreement of the acceleration curve shape, the duration of the signal build up, and replicates the decrease of acceleration.

Evolving from the simplified ellipsoidal head model presented in Figure 5a, the advanced impact simulations concerning the viscoplastic weakening material, were run for the complex head–brain model presented in Figure 5b, which was created based on images acquired from medical scanners and discretised into tetrahedral spatial elements [45].

The presented example has a complexity of 15,596 nodes (46 k variables) and 50,796 elements describing the brain, and was solved 1.2 million times for 12 ms of the simulation period. This model was dedicated to obtaining accurate acceleration mappings, while the simplified model allowed efficient parameter identification prior to high-resolution simulations. It would be computationally inefficient to run a minimisation procedure using a high-resolution model or perform acceleration mapping for the low-detailed model. A viscoplastic weakening material with *m* = −0.5, *s* = 1.2, *K* = 20 Pa s, *c* = 12 Ns/m was assumed and large deformations of the tissue were taken into account.

The conversion of the geometrical model to the space–time finite element discrete system was carried out by customised computer code, to allow for parallel computations. The time needed for a single pass of the impact simulation using parallel computing is shorter almost by a factor of 25, compared to using a serial execution.

**Figure 9:**
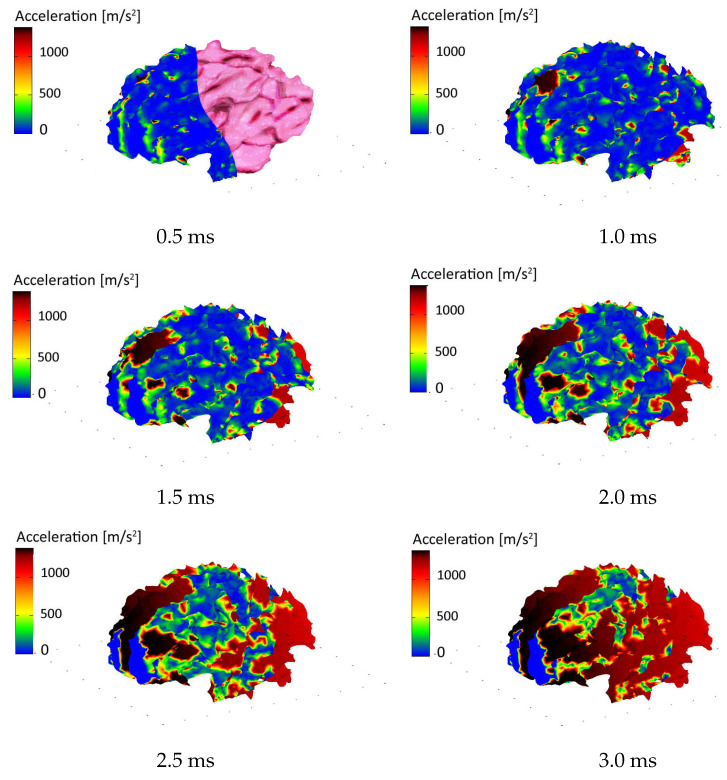
Simulated accelerations in the brain at six successive stages of head impact.

Figure 9 presents six consecutive frames from the brain impact simulation, showing precise regions of high acceleration. The outer skull boundary layers were removed from the figures for better transparency of the results.

At the selected point of the brain, the inner points exhibit acceleration reaching over 1000 m/s${}^{2}$, making this area of the brain most vulnerable to injuries related to exceeding critical strain. The viscoplastic softening model can replicate the impact dynamics with fair accuracy concerning the acceleration values and duration of the process. One should strive to design solutions shifting the recorded values below the so-called death line, i.e., the curve above which the brain is subjected to too high forces for too long, making the damage irreversible. Detailed insight into the state of selected sensitive brain zones will allow better design of passive head protection.

## 5. Conclusions

The problem of simulating the deformation of cerebral structures was considered and a new viscoplastic model suitable for the simulation of mechanical behaviour in the brain, during rapid excitation related to impact events, was introduced. The system’s response is devoid of hyperbolic effects favouring purely parabolic ones. The model might be adapted to simulate other soft materials like gels, jellies, thick liquids, or even fluid–structure interactions. Nevertheless, one has to be aware that the proposed model is valid just for the initial phase of impact response, when a rapid increase in accelerations and decelerations dominate. In contrast, the successive phases, which involve decreasing velocity and oscillatory motion, might benefit from incorporating viscoelastic behaviour. The problem of obtaining an accurate solution in a reasonable time was solved with a parallel implementation of a space–time discretisation method, instead of using a standard space discretisation approach. This allowed the assessment of mechanical parameters in the brain tissue caused by the impact of mechanical loads and comparing the results with experiments in the literature.

These findings demonstrate the insufficiency of existing viscoelastic models as a primary mechanical characterisation for brain mimetic materials and provide quantitative information for the future design of materials that more closely replicate mechanical features of the brain. Further research plans concern simulating the interaction of the brain and the skull, including the soft outer layers, to allow designing an effective system protecting the head from consequences of violent impacts. 

## Figures and Tables

**Table 1:** List of material cases: Constant (*m* = 0.5) and softening matter (*m* = −0.5).

Case	Coefficient *s*	Acceleration [m/s${}^{2}$]
case with *m* = 0.5	—	2750
*m* = −0.5, case A	2.00	1260
*m* = −0.5, case B	1.00	1070
*m* = −0.5, case C	0.50	1030
*m* = −0.5, case D	0.20	650
*m* = −0.5, case E	0.05	470

## Data Availability

The data presented in this study are available on request from the corresponding author.
